# Dietary Effects of Anthocyanins in Human Health: A Comprehensive Review

**DOI:** 10.3390/ph14070690

**Published:** 2021-07-18

**Authors:** Ana C. Gonçalves, Ana R. Nunes, Amílcar Falcão, Gilberto Alves, Luís R. Silva

**Affiliations:** 1 CICS–UBI—Health Sciences Research Centre, University of Beira Interior, 6201-506 Covilhã, Portugal; anacarolinagoncalves@sapo.pt (A.C.G.); araqueln@gmail.pt (A.R.N.); gilberto@fcsaude.ubi.pt (G.A.); 2 CIBIT—Coimbra Institute for Biomedical Imaging and Translational Research, University of Coimbra, Edifício do ICNAS, Pólo 3, Azinhaga de Santa Comba, 3000-548 Coimbra, Portugal; vr.amilcar.falcao@uc.pt; 3 Laboratory of Pharmacology, Faculty of Pharmacy, University of Coimbra, Pólo das Ciências da Saúde, Azinhaga de Santa Comba, 3000-548 Coimbra, Portugal

**Keywords:** anthocyanins, antioxidants, dietary source, bioavailability, biological activity, biosynthesis

## Abstract

In recent years, the consumption of natural-based foods, including beans, fruits, legumes, nuts, oils, vegetables, spices, and whole grains, has been encouraged. This fact is essentially due to their content in bioactive phytochemicals, with the phenolic compounds standing out. Among them, anthocyanins have been a target of many studies due to the presence of catechol, pyrogallol, and methoxy groups in their chemical structure, which confer notable scavenging, anti-apoptotic, and anti-inflammatory activities, being already recommended as supplementation to mitigate or even attenuate certain disorders, such as diabetes, cancer, and cardiovascular and neurological pathologies. The most well-known anthocyanins are cyanidin 3-*O*-glucoside and cyanidin 3-*O*-rutinoside. They are widespread in nature, being present in considerable amounts in red fruits and red vegetables. Overall, the present review intends to discuss the most recent findings on the potential health benefits from the daily intake of anthocyanin-rich foods, as well as their possible pharmacological mechanisms of action. However, before that, some emphasis regarding their chemical structure, dietary sources, and bioavailability was done.

## 1. Introduction

In the past few years, it is widely accepted that the daily intake of medicinal herbs, fruits, and vegetables provides a wide array of benefits to human health [1]. This fact is essentially due to their composition, plentiful in several non-nutrient bioactive compounds, such as phenolics, whose abilities to modulate different processes and pathways in the human body, such as regulating glucose levels and boosting antioxidant, anti-inflammatory, anti-mutagenic, anticancer, and neuroprotective effects, are already well-known [2,3].

Considering that nature-based products have been used since ancient times to treat several disorders, such as colds, pain, gastrointestinal aches, and hypertension, among others, it is not surprising that this tendency continues to increase worldwide [4]. A clear example is their incorporation into pharmaceutical products used in cancer therapy [5,6]. According to the database of 2019, from the 247 anticancer drugs available in the market, about 81.0% are derived from natural products; the remaining ones are synthetic drugs (15.3%) or vaccines (3.65%) [3]. 

Among the phenolic compounds, anthocyanidins and their conjugated acyl-glycosylated or glycosylated forms, called anthocyanins, are both members of the flavonoids and an interesting class of water-soluble vacuolar pigments [7]. They are synthetized via the flavonoid path and considered the major contributors to the vivid red, orange, violet, and blue colours exhibited by various edible flowers, vegetables, fruits, some cereals, seeds and plant leaves, and their derivatives, such as juices, tea, and red wines [8]. They also have received much attention owing to their nutritional value, pharmacokinetic profile, pharmacological mechanisms, and health-promoting properties [9,10]. Indeed, recent human and animals surveys revealed that they are functional compounds able to increase antioxidant defences, diminish free radical damage, chronic inflammation and the risk of mutations, and attenuate, or even mitigate, the development and progression of many non-communicable and degenerative chronic disorders, namely, atherosclerosis, metabolic syndrome, eye and kidney complications, many cancer types, and also to control weight [6,11,12,13,14,15,16,17,18,19]. These biological activities are associated with their chemical structure, the presence of the catechol and pyrogallol groups standing out, allowing them to have the ability to chelate metal ions and neutralize free radicals and reactive species [4,20,21,22]. The predominant ones found in foodstuffs are cyanidin, delphinidin, pelargonidin, peonidin, petunidin, and malvidin glycosides [6,23,24]. 

This comprehensive review aimed to assess and elucidate the impact of anthocyanins and anthocyanin-rich foods on human health. For that, the first part of the manuscript focuses on a description regarding the chemical structure and function as well as main dietary sources of anthocyanins. Afterwards, the bioavailability and metabolism after intake are mentioned. Finally, we summarized and discussed the most recent literature regarding the main therapeutic effects of anthocyanins on different disorders.

## 2. Chemical Structure and Function of Anthocyanins

**Figure 1:**
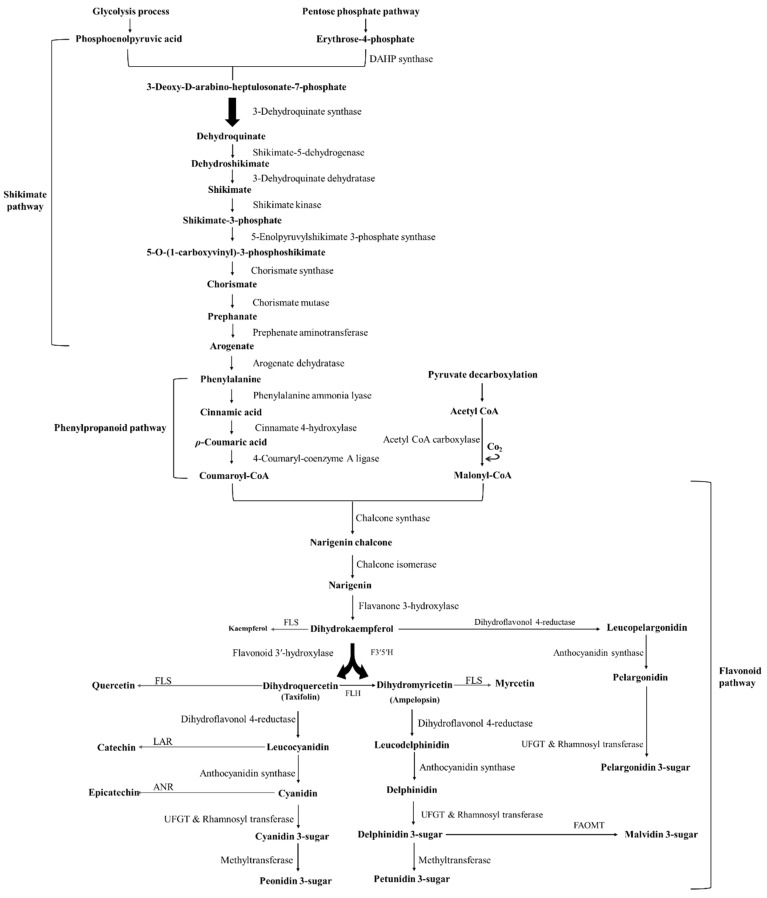
Biosynthesis pathways of the main anthocyanins found in foods. DAHP: 3-Deoxy-D-arabinoheptulosonate 7-phosphate; CoA: coenzyme A; F3′5′H: Flavonoid 3′, 5′-hydroxylase; FLH: Flavanone 3-hydroxylase; FLS: Flavonol synthase; LAR: Leucoanthocyanidin reductase; ANR: Anthocyanidin reductase; UFGT: UDP glucose flavonoid 3-*O*-glucosyltransferase; FAOMT: Flavonoid 3’, 5’-methyltransferase (adapted from [25]).

Phenolic compounds are secondary metabolites produced by plants to protect them against pathogens and predators, ultraviolet radiation, climate conditions, and acidified soils, acting also as attractants for pollinators, antifeedants, and phytoalexins [25,26]. They are also considered the main contributors to plants’ colour, nutritional, and sensory characteristics [27]. Their structure presents at least one benzene ring coupled to one or more hydroxyl groups and can range from simple phenolic, low molecular weight and single-aromatic molecules to highly polymerized compounds [28]. In order to facilitate their distinction, phenolics are classified into two major groups: (i) non-flavonoid compounds (phenolic acids, tannins, lignans, coumarins, stilbenes, and curcuminoids) and (ii) flavonoid compounds (anthocyanidins, flavan-3-ols, and their oligomeric structures, recognized as proanthocyanidins, flavanones, flavanonols, flavones, flavonols, and isoflavones) [7,25,26]. Their biosynthesis, which is shown in Figure 1, comprises the shikimate, phenylpropanoid, and flavonoid pathways, and involves deamination, hydroxylation, and methylation reactions [25]. 

**Figure 2:**
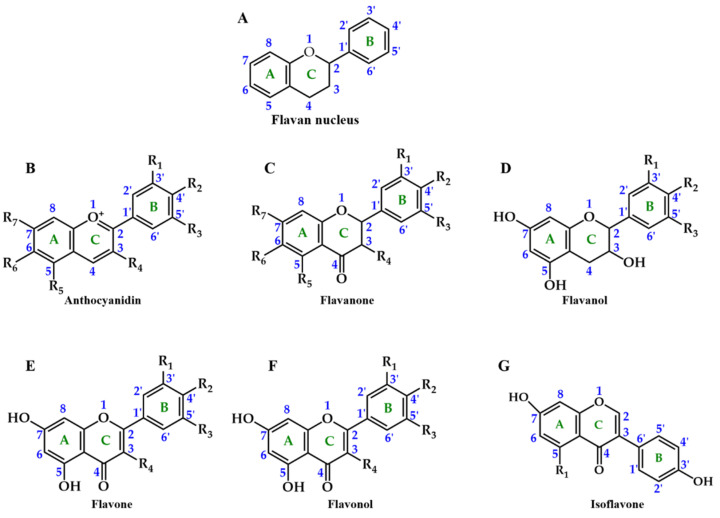
The basic structure of the flavonoids (**A**), which include anthocyanidins (e.g., cyanidin and pelargonidin) (**B**), flavanones (e.g., hesperidin and naringenin) (**C**), flavanols (e.g., catechin and epicatechin) (**D**), flavones (e.g., apigenin and luteolin) (**E**), flavonols (e.g., quercetin and kaempferol) (**F**), and isoflavones (e.g., daidzein and genistein) (**G**), differing in the level of oxidation and C ring saturation (adapted from [31]).

Concerning the flavonoids (Figure 2), they represent about two-thirds of the total dietary phenolics consumed, and currently, more than 9000 different flavonoids have been identified so far [29,30]. They all possess a common flavan nucleus, i.e., a 15 carbon-structure (C6 (A ring)-C3 (C ring)-C6 (B ring)), composed of 2 aromatic rings (A and B rings derived from the acetate/malonate and shikimate pathways, respectively), linked by a heterocyclic benzopyran 3-carbon ring that contains an oxygen atom (C ring) [26,31]. However, they differ in (i) the C ring unsaturation and oxidation state; (ii) A, B, and C ring substituents, such as the presence or absence of double bonds and carbonyl groups, and the possible occurrence of acylation, alkylation, glycosylation, oxygenation, and sulphonation processes; (iii) the position where the B ring is linked to the C ring; and (iv) location and number of hydroxy and methoxy groups on the B ring [26].

**Figure 3:**
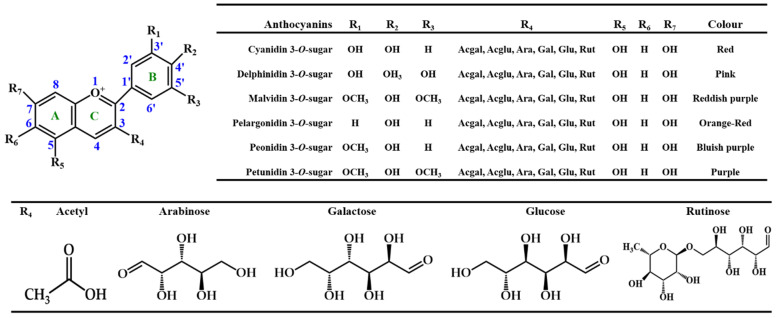
Representation of the main general chemical structure of anthocyanins. Acgal—acetylgalactose; Acglu—acetylglucose; Ara—arabinose; Gal—galactose; Glu—glucose; Rut—rutinoside (adapted from [35,37]).

Focusing on anthocyanidins, their B ring is joined to the C ring through carbon 2 and the sugar residue of anthocyanins is typically found conjugated at carbon 3 [32,33] (Figure 3). Furthermore, their capacity to create flavylium cations makes them distinguishable from other flavonoid subclasses [22]. To date, about 27 different structural anthocyanin aglycones and 1000 anthocyanins are known [34,35]. In foods, the most commonly found are sugar moieties of cyanidin (50%), which is the most studied given their large distribution and number of hydroxyl groups, followed by pelargonidin, peonidin, and delphinidin (12%), and finally by petunidin and malvidin glycosides (7%) [8] (Figure 3). Together, they represent 90% of the total anthocyanins identified until now [36]. Their structure directly influences their biological potential, namely, the number of hydroxyl groups, the degree of glycosylation and acylation, the catechol residue on the B ring, and the oxonium ion on the C ring [37]. 

Therefore, the classification of anthocyanins is made according to (i) the number, position, and degree of methylation of the hydroxyl groups; (ii) the number and nature of the sugar moieties bonded to the aglycone; and (iii) the position of the aliphatic and/or aromatic carboxylate acids on the sugar molecule [38]. The stability of the anthocyanins is usually affected by the storage and processing conditions, temperature, and cooking, as well as by exposure to light and oxygen, the presence of enzymes, other phenolic compounds, metal ions, ascorbic acid, hydrogen peroxide (H_2_O_2_), water, and/or sulphites [37].

## 3. Major Sources of Anthocyanins

Anthocyanins are widely spread in nature and are considered mainly responsible for the vibrant red, blue, and purple colours exhibited by vegetables, fruits, and their derivatives [37,39]. Their levels differ markedly among different species, being largely influenced by plant genotypes, and less by agricultural practices, growing area, climatic conditions, seasonal variability, temperature and light exposure, ripening stage, harvest time, and the adopted methods for processing and storage [9,40].

The daily average intake of anthocyanins is estimated to vary from several milligrams to hundreds of milligrams, although its evaluation is inaccurate and depends on the diet, gender, existence (or not) of food intolerances in individuals, and their quantities in foods [41]. Their intake by humans is higher in countries with a Mediterranean diet, plentiful in reddish berries and other red and blue-coloured fruits and vegetables, and red wine [23,42]. In Europe, the ingestion ranges from 19.8 mg per day (the Netherlands) to 64.9 mg per day (Italy) in men and 18.4 mg per day (Spain) to 44.1 mg per day (Italy) in women [24]. In the United States of America, Australia, and Asian countries, the intake is about 12.5, 24.2, and 37 mg per day per person, respectively [23,42,43]. Furthermore, and although it is not well established since they are non-essential nutrients, the recommended daily consumption of these coloured compounds has been evaluated, with China already recommending a daily intake of 50 mg per day/person in order to reduce oxidative stress levels and consequently the risk of cancer, metabolic syndrome, diabetes, degenerative diseases, and other pathologies [44]. 

In a general way, the primary sources of anthocyanins are berries (43% in Europe and 39% in the USA), red wine (22% in Europe and 18% in the USA), vegetables, and other fruits (19% in Europe and 9% in the USA) [45]. Table 1 presents the main sources of anthocyanins. A major amount of anthocyanins are found in berries, especially elderberries, chokeberries, blueberries, pomegranate, and açaí, presenting values superior to 282.5 mg cyanidin 3-glucoside equivalent per 100 g of fresh product [46,47]. Among the anthocyanins, cyanidin, malvidin, and delphinidin glycosides are the most found [47,48,49,50]. 

## 4. Anthocyanins’ Bioavailability and Metabolism 

Understanding the bioavailability of phenolics is crucial since, after consumption, their constituents undergo many modifications throughout the digestive tract (digestion, absorption, metabolization, and elimination), which have a great impact on their nutritional value and health-promoting properties [28,31]. 

Nowadays, it is established that the bioavailability of phenolics differs significantly between them, and therefore, the most abundant polyphenols in our dietary habits are not necessarily those that show the highest concentrations of active metabolites in organs and tissues [35]. Indeed, their bioavailability is highly dependent on the chemical structure of the phenolics, i.e., their molecular size, pattern of glycosylation and/or acylation (where acylation increases anthocyanin stability but declines their absorption), degree of polymerization, and conjugations and/or combinations with other compounds [36,91]. Furthermore, it is also influenced by the pH values observed along the gastrointestinal tract, facilitation of the compounds to cross membranes, digestibility, solubility, and absorption actions carried out by digestive enzymes, biliary acids, gut microbiota, and enterocytes. The food matrix maturity degree and cooking also influence the availability rates [35]. Although the thermal processing reduces anthocyanins’ stability, at the same time, it damages the anthocyanin cell walls, increasing their body absorption [28]. 

It is also important to take into account that the bioavailability also varies among individuals—inter-individual variability—due to intrinsic aspects (e.g., age, sex, physiological and/or pathological states, and genetic factors), which induce marked differences regarding enzymes and microbiota activity [43]. 

Unlike other phenolics, anthocyanins are quickly metabolized and eliminated, and even in high amounts, they rarely reach concentration values considered active. In fact, the intake of 10 and 720 mg of anthocyanins only results in maximal plasma concentrations of 1.4 and 200 nanomolar, respectively, achieved between 30 min and 2 h [92,93,94]. 

Table 2 summarizes the principal human trials focused on the pharmacokinetic profile of anthocyanins observed after their ingestion in common foods and beverages. In a general way, less than 1.8% of consumed anthocyanins is normally absorbed. However, this percentage can decrease if they are ingested alone, with or after other compounds; i.e., if anthocyanins are consumed alone or after overnight fasting, their digestion happens in 1 hour [93,95], whereas if they are ingested accompanied by other foods and beverages, ingested with high-fat meals, or after a meal, it occurs only after 1.5 or 4 h, respectively [96]. They disappear from the blood circulatory system in less than 6 h [57].

Additionally, and considering that substitutions influence anthocyanin absorption, nowadays, it is well-described that pelargonidin derivatives are more readily absorbed than anthocyanins with more substituents on their B ring, such as peonidin-, delphinidin-, and cyanidin-based anthocyanins [46]. Furthermore, and comparing the sugar moieties, it was already verified that malvidin 3-*O*-arabinoside presents higher absorption rates than malvidin 3-*O*-glucoside [103]. 

Despite their low absorption and rapid metabolism, the regular consumption of anthocyanins is considered safe, and together with physical activity, it is encouraged as both can reduce the occurrence of several pathologies related to oxidative stress [36]. As far as we know, until now, no adverse effects regarding anthocyanin consumption have been reported. In fact, and focusing on human studies, most people who consumed 160 mg of anthocyanins twice a day for 2 months tolerated the extract; only 4% of the participants revealed side effects, namely at the gastrointestinal level and eczema [104,105].

Therefore, after being consumed, anthocyanins are metabolized in the mouth, where the action of oral microbiota removes the glycosidic groups and transforms them into their corresponding chalcones [106]. 

After that, they pass along the gastrointestinal tract, starting in the stomach, where they do not suffer considerable changes, despite the acidic pH, and can be absorbed by bilitranslocase, becoming available for absorption (bioaccessibility) [106,107], or reach intestinal epithelial cells [108]. In fact, the literature suggests that it is possible that the gastric and intestinal bioavailability of anthocyanins are mainly done with the 3-monoglucosides, 3-monoglucoside acylated, and 3,5-diglucosides forms [106,107,109].

**Figure 4:**
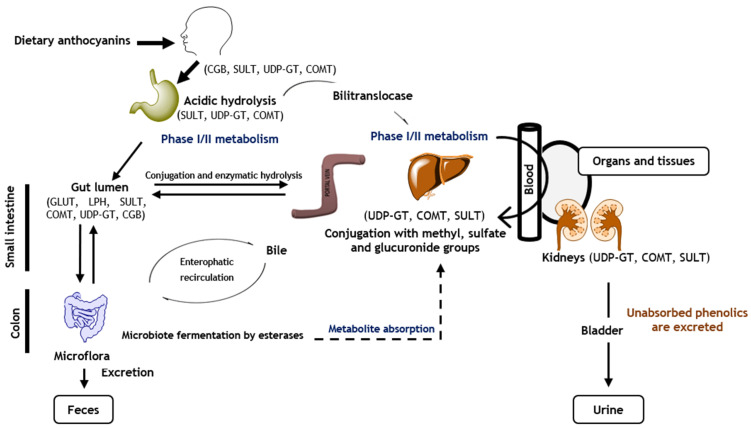
Anthocyanin absorption, distribution, metabolism, and excretion. CGB—cytosolic *β*-glucosidase; SULT—sulfotransferase; UDP-GT—glucuronosyltransferase; COMT—catechol-*O*-methyl transferase; GLUT—glucose transporters; LPH—lactase-phlorizin hydrolase.

Therefore, anthocyanins can go through the portal vein to the liver and can be directed to the systemic circulation to be taken up by the target organs and tissues, or, if they are not absorbed, be discarded through urine and faeces (Figure 4) [41,110]. It is important to note that in intestines, liver, and kidneys, anthocyanins are metabolized by enzymes of phase I and phase II, being conjugated with additional hydroxyl, methyl, sulfuric, or glycoside groups in order to increase their availability [33,36,111]. 

In general, both native anthocyanidins and their conjugated forms are found in plasma and urine; nevertheless, the intact ones are more rapidly absorbed in the stomach (20–25%) and detected in plasma a few minutes after their ingestion [112]. This evidence is supported by previous studies based on the oral ingestion of red fruits, which revealed that anthocyanins are not metabolized into their aglycones, being directly absorbed and appearing in plasma 30 min after their consumption [92]. Their glycosylated forms are excreted in urine [8,93]. 

Notwithstanding, the major absorption rate is observed in the gut [33]. Therefore, in the gut, the lactase-phlorizin hydrolase (LPH) and *β*-glucosidase enzymes release the aglycone of the coloured compounds, increasing their hydrophobic character, thus facilitating their entrance by passive diffusion in epithelial cells [113]. Glycosides and acylated anthocyanins can also be absorbed by the small intestine due to the action of glucose transporters 1 and 3 (GLUT 1 and 3) [91,109,111,114]; however, the absorption of the acylated ones is four times lower than that of non-acylated anthocyanins [33]. Particularly, molecular docking studies revealed that smaller molecules interact with GLUT 1 and 3 by their glucose residue and AC rings, while larger compounds penetrate in both transporters by their C5 glucose, as well as B and coumaroyl rings [91,109].

Unabsorbed anthocyanins reach the colon and are hydrolysated within 20 min−2 h by colonic bacteria (e.g., *α*-galactosidase, *β*-D-glucuronidase, *β*-D-glucosidase, and *α*-rhamnosidase), which break down the glycosidic bonds and catalyse them into smaller phenolic compounds (e.g., benzaldehydes or hydroxytyrosol) or simple phenolic acids, such as *ρ*-hydroxybenzoic, homovanillic, phenylpropionic, protocatechuic, syringic, and vanillic acids, to simplify their absorption by colonic mucosa and consequently increase their availability [115]. This extensive metabolization is tremendously interesting and shows that the availability of anthocyanins is probably higher than we thought, and the reason why anthocyanins can be found in urine in amounts below 0.1% [91,116]. Indeed, a recent study indicates the intake of 300 g of red raspberries results in the identification of 18 different anthocyanin-derived metabolites [77]. Basically, delphinidin-based anthocyanins are transformed into 2,4,6-trihydroxybenzaldehyde, gallic, and syringic acids, while syringic, 4-hydroxybenzoic, and vanillic acids are the primary metabolites of malvidin, pelargonidin, and peonidin glucosides, respectively [111,117]. Cyanidin glycosides can produce around 35 metabolites, 31 being found in urine samples, 28 in faeces, and 17 in the circulatory system, where the main ones are 2,4,6-trihydroxybenzaldehyde, *ρ*-coumaric, protocatechuic and vanillic acids, and phenolic conjugates (e.g., hippuric, phenylacetic, and phenylpropenoic acids) [77,118]. 

Additionally, the cleavage of glycosidic bonds also enhances the beneficial effects exhibited by anthocyanins [33]. In fact, this modulation on colon microflora leads to the formation of short-chain fatty acids that together with phenolic acids induce a decrease in pH values, creating favourable conditions for the proliferation of probiotic bacteria, such as *Actinobacteria, Bifidobacteria*, and *Lactobacilli* [33,111]. These bacteria exert positive effects in the control of gastrointestinal and digestive disorders, allergies, eczema, and improvements in delicate cases of cardiac and mental illness [10,119].

## 5. Anthocyanin Encapsulation 

Knowing that the incorporation of phenolic compounds into foods and pharmaceutical products is a challenge, due to their instability and susceptibility to degradation, during processing and storage, various delivery systems have been developed [4]. Among them, encapsulation is a good strategy. This technology allows entrapping an active agent, liquid, gas, or solid within a matrix or a polymeric wall in micro or nanoparticles, to protect the active compound from environmental conditions, undesirable interactions, and to control their transportation, release, and handling. The most common polymers used are carbohydrates (e.g., cellulose derivatives and maltodextrins), natural gums (e.g., alginates and gum arabic), lipids (e.g., emulsifiers and waxes), and/or proteins (e.g., dairy proteins, gelatine, and soy proteins) [33]. Additionally, their combination with other wall materials and some modifiers, such as antioxidants, chelate agents, and surfactants, can also increase the encapsulation benefits [4]. In a general way, the process of encapsulation is based on the formation of the wall around the compound of interest, ensuring that unwanted materials are kept outside, and preventing undesired leaks to happen. It is important to take into account, along with its cost, the particle size and physicochemical properties of the core and the origin of wall constituents, to favour capsule stability. To that end, several methods have been developed, and the best-known ones for anthocyanin encapsulation are emulsification, ionotropic gelation, thermal gelation, and spray-drying. This last one is the most applied technique (80–90% of encapsulated formulations are spray-dried) due to their cost and easy procedure [4]. Maltodextrins are the most used coating material given their ability to maintain anthocyanin stability [120].

Therefore, and in order to enhance their bioefficacy and stability, and thus prevent their rapid degradation, anthocyanins are mainly encapsulated with liposomes, nanocomplexes of alginate and chitosan, and gel emersions [34]. In fact, several studies already showed that anthocyanin nano-formulations, along with chemical modifications, favour their absorption and metabolization, and consequently increase their biological action [33]. Mueller et al. [112] conducted a human study and reported that the encapsulation of 2.4 g of blueberry anthocyanins with whey protein does not contribute to anthocyanin stabilization during intestinal passage given their ability to prolong their passage through the stomach, whereas the encapsulation with citrus pectin improves anthocyanin bioavailability and intestinal accessibility, thus increasing their concentrations in the bloodstream.

Furthermore, bioengineering-based, targeted drug delivery approaches using biodegradable PLGA@PEG nanoparticles revealed more notable results in both in vivo and in vitro Alzheimer’s disease models than anthocyanins alone, namely, lower levels of oxidative stress and neuroinflammatory hallmarks [121,122]. Furthermore, 50 mg/mL of blueberry anthocyanins encapsulated in liposomal micelles also revealed higher anticancer activity than non-encapsulated anthocyanins on K562 human erythroleukemic cancer cells [123].

## 6. Putative Health Benefits

**Figure 5:**
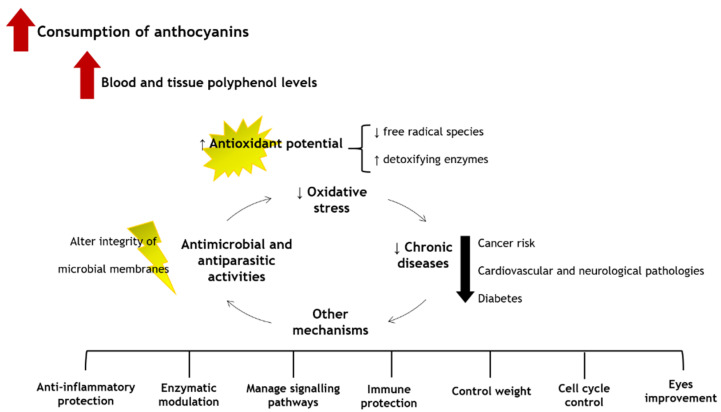
Health benefits of anthocyanins.

The low ingestion of fruits and vegetables causes around 1.7 million deaths worldwide, being related to 14% of gastrointestinal malignancies, 9% of stroke, and 11% of coronary artery disorders [36]. Therefore, it is undeniable the role of anthocyanins in promoting human health and welfare [105]. Several in vitro scavenging assays, animal and human cell lines studies, animal models, and human clinical trials already indicated that the consumption of foods, beverages, and supplements rich in anthocyanins brings numerous health benefits. In fact, this is due to the easy capacity of the anthocyanins to eliminate and/or neutralize free radicals and reactive species, chelate metals, control signalling pathways, diminish pro-inflammatory markers, and, thus, reduce the risk of cardiovascular pathologies, cancer, and neurodegeneration. Additionally, they also contribute to control weight and improve vision. The general health benefits resulting from the consumption of anthocyanins are shown in Figure 5.

### 6.1. Antimicrobial Effects and Anti-Parasitic Activity

In order to attenuate the emerging resistance of microorganisms to antibiotics over time, natural products have gained much attention since they are rich in many metabolites with antimicrobial, antifungal, and anti-parasitic effects. In fact, these activities are part of the plants’ defense mechanism against pathogens and infections during their development and growth. Among these phytochemicals, anthocyanins already showed capacity to reduce the replication and growth of some Gram-negative and Gram-positive bacteria and parasites [124,125].

Anthocyanins already revealed an ability to stop the replication of two common foodborne pathogens, *Escherichia coli* and *Salmonella*, exhibiting minimum inhibitory concentration (MIC) values varying from 10 to 400 mg/mL, and also to reduce the growth of *Desulfovibrio s*pp. and *Enterococcus* pathogenic bacteria by increasing the abundance of probiotics, such as *Akkermansia* and *Bifidobacteria* [124]. These results are in accordance with those reported by Sun et al. [125], who also revealed that anthocyanin-rich extracts from blueberries can also destroy the cell membrane of *Listeria monocytogenes, Staphylococcus aureus*, and *Vibrio parahaemolyticus*, displaying MIC scores of 0.27, 0.21, and 0.030 mg/mL, respectively, after 24 h of exposure. Furthermore, it has also been reported that 100 µM cyanidin 3-*O*-glucoside can also inhibit the expression and secretion of *Helicobacter pylori* toxins by suppressing the SecA transcription pathway, thereby making the proteins’ exportation difficult [126]. Even though delphinidin 3-*O*-rutinoside and cyanidin 3-*O*-rutinoside at 1% did not reveal any inhibitory effect, enriched fractions of berries at concentrations ranging from 0.1 to 1% showed capacity to inhibit the yeasts *Saccharomyces cerevisiae* and *Rhodotorula rubra*, and the bacteria *Bacillus cereus*, *Salmonella typhimurium*, and *Lactococcus lactis* cocci growth [127]. Mulberry anthocyanins also showed potential to interfere with the development of *Pseudomonas aeruginosa*, a microorganism associated with biofilm-mediated infections, exhibiting an MIC value of 2 mg/mL [128]. Similar results were observed concerning the anti-microbial effects of blackcurrants and cherries on suppressing the growth of *S. aureus, E. coli*, and *P. aeruginosa* bacteria [129]. More recently, Silva et al. reported that anthocyanin-rich blueberry extracts at concentrations superior to 1 mg/mL can also inhibit virulence factors, namely, the formation of biofilms and adhesion of *Acinetobacter baumannii* and *Proteus mirabilis* pathogenic microorganisms over 24 h of treatment [130].

A dose of 0.225 µg/mL of cornelian cherry fruits showed potential to stimulate murine immune response during *Trichinella spiralis* infection, a causative agent of human trichinellosis, by enhancing the CD3^+^, CD4^+^, and erythrocytes cells, promoting platelet aggregation and decreasing CD8^+^ splenocyte cells when compared to *T. spiralis*-infected mice that did not receive the extract [131]. Besides, it has already been reported that Kenyan purple tea anthocyanins (200 mg/kg) can ameliorate post-treatment reactive encephalopathy associated with cerebral human trypanosomiasis, caused by *Trypanosoma brucei* parasites in a murine model, after 21 days of treatment, by delaying the establishment of the trypanosomes and increasing the glutathione and aconitase-1 levels in the brain compared to the untreated group [132]. Additionally, anthocyanins isolated from black soybean already revealed the potential to control chronic bacterial prostatitis, an infection from the lower genitourinary tract, in rat models, over 4 weeks of treatment at doses of 50 mg/kg administrated twice a day for 2 weeks compared to the control group, which did not receive anthocyanins [133]. 

In a general way, the antimicrobial, antifungal, and anti-parasitic activities of the anthocyanins are mainly due to their capacity to react with the DNA, proteins, and sulfhydryl groups and interfere with AKT, ATPase, and superoxide dismutase activities, which, in turn, decrease the citric acid cycle and microbial metabolism, and inhibit microbial enzymes [125,132]. These events deprive microorganisms of the substrate that they need, compromising their development and replication, the formation of biofilms and host ligand adhesion, and lead to cytoplasmatic membrane destabilization and consequent cell death.

### 6.2. Antioxidant Properties

Free radicals are a product of natural metabolism; however, their accumulation becomes toxic to cells and trigger many reactions, such as the oxidation of cellular components (nucleic acids, proteins, and fatty acids) and lipid peroxidation, accelerating aging processes, and eliciting the occurrence of many chronic diseases, such as neurodegenerative and cardiovascular disorders, cancer, atherosclerosis, and ulcerative colitis [4]. These reactive species can be derived from oxygen (e.g., hydroxyl, peroxyl, and superoxide) or nitrogen (e.g., nitric oxide and peroxynitrite). Besides, there are also even-numbered free radical species, such as H_2_O_2_ and lipid peroxide. Since synthetic antioxidants have various adverse effects on health, there is a trend towards relying on antioxidants from natural products. Currently, the notable antioxidant properties of anthocyanins are well-described, and it is mainly due to their conjugated rings and hydroxyl groups [41,134,135]. 

Many in vitro assays already demonstrated the antioxidant potential of anthocyanin derivatives and foods rich in them [136]. Focusing on individual anthocyanins, Rahman et al. [137] reported that delphinidin isolated from blueberry extracts shows the most considerable capacity to scavenge superoxide species, followed by petunidin > malvidin = cyanidin > peonidin > pelargonidin, at 1 µM. Similar results were obtained, regarding the capacity of these compounds, at the same concentration, to capture peroxynitrite radicals [133]. Moreover, cyanidin 3-*O*-glucoside at concentrations between 100 and 200 µM showed potential to protect human keratinocyte HaCaT cells against ultraviolet-A radiation, preventing DNA fragmentation and the release of hydrogen peroxide (H_2_O_2_) [138].

Regarding the anthocyanin-enriched fractions from natural products, extracts of blackberries, blueberries, strawberries, sweet cherries, and red raspberries at 10 µM displayed the potential to inhibit human LDL oxidation, having been two times more effective than an ascorbic acid control [139]. The coloured fraction of the sweet cherries also showed capacity to scavenge nitric oxide, in a concentration-dependent manner, displaying a half-maximal inhibitory concentration (IC_50_) value of 47.44 µg/mL, being three times more active than ascorbic acid (IC_50_ = 179.69 µg/mL); it also protects human erythrocytes against haemoglobin oxidation and prevents haemolysis damage induced by peroxyl radicals, in a concentration-dependent manner (IC_50_ = 33.86 and 9.44 µg/mL, respectively). Positive correlations (r > 0.4) between anthocyanin content and antioxidant assays have been reported [134]. Additionally, the same extract also exhibited potential to capture superoxide species in a concentration-dependent manner (25% inhibitory concentration (IC_25_) score of 16.58 µg/mL) and protect human adenocarcinoma Caco-2 cells against oxidative stress induced by *tert*-butyl hydroperoxide [130]. Blackberries extracts rich in anthocyanins revealed a ferric-reducing antioxidant power score of 191 µmol Fe^2+^/L (extract concentrations between 10 and 200 µg/mL) [138] and also capacity to quench peroxyl radicals (4885 µmol Trolox equivalent/g) [140]. After 24 h of treatment, the same extract, at concentrations varying between 0.02 and 50 µg/mL, also showed an ability to protect human intestinal INT-407 normal cells against intracellular oxidation induced by 2,2’-azobis(2-amidinopropane) dihydrochloride (IC_50_ = 4.1 µg/mL) [140]. Blackberry and raspberry fruits also revealed lipid peroxidation inhibitory potential, displaying IC_50_ values lower than 50 µg/mL [141]. 

Concerning in vivo trials, delphinidin (1 mg/0.1 mL DMSO/mouse) showed capacity to protect mouse skin against UV-B radiation, preventing apoptosis after 8 h of exposure [142]. Additionally, rats that were fed during 35 days with anthocyanin extracts (4 mg/kg of body weight) from blackberries showed a meliorate antioxidant status compared to animals that did not receive these phenols in the diet, with lower levels of reactive species in the brain, liver, kidney, and plasma standing out (−35%, −44%, −17% and −8%, respectively), as well as higher catalase and glutathione peroxidase concentrations in the brain, kidney, and liver (+0.30, +0.65 and +0.05%, respectively) [143]. Anthocyanins from dabai fruits also showed potential to increase superoxide dismutase action (+10%) and to inhibit lipid peroxidation (−21%) in white rabbits that ingested 2000 mg/day of the extract for 8 weeks, compared to the control group. These benefits are attributed to the ability of anthocyanins to disrupt the activity of poly(ADP-ribose) polymerase 1 [144]. 

In humans, the ingestion of fresh strawberry fruits (300 g, possessing 9.57 mg of anthocyanins) by 13 healthy volunteers revealed the capacity to increase the plasma ferric-reducing antioxidant power (+3.1%), *α*-carotene (+7%), and vitamin C (+23%) levels [145]. Besides, 12 healthy participants who consumed açaí juice and pulp, composed of 165.9 mg/L and 303.8 mg/kg of anthocyanins, respectively, showed increments in plasma antioxidant capacity of 3- and 2.3-fold, respectively [11]. Furthermore, forty-seven healthy adults who consumed 30 mL of tart cherry concentrate diluted to a volume of 250 mL with water for 6 weeks showed higher levels of plasma ferric-reducing ability than the control group (+10%) [12]. A randomised cross-over study involving 30 healthy female participants who drank 330 mL of an anthocyanin-rich beverage over 14 days displayed increases in superoxide dismutase activity of about 6%, compared to the placebo group [146]. More recently, 300 g of blueberries ingested by ten young volunteers showed potential to protect human blood mononuclear cells against oxidative damage induced by H_2_O_2_ compared to the control, by reducing oxidative damage by 18% after 24 h of their consumption [147]. The same was verified after a 30-day-treatment with 500 g of strawberry fruits [148]. Anthocyanin-rich juices and nectars also showed potential to aid recovery after strenuous exercise by increasing the plasma total antioxidant capacity and diminishing lipid peroxidation and carbonyl species [149,150,151].

These considerable health-promoting properties are intimately linked to the capacity of anthocyanins to increase the glutathione levels by restoring or raising the activity of exogenous antioxidant enzymes and by activating the genes responsible for coding these enzymes. Besides, it is also due to their ability to inhibit NADPH and xanthine oxidases and modify arachidonic metabolism and mitochondrial respiration, and hence, reduce the formation of free radicals and reactive species, protecting cell components from damage [41]. Furthermore, anthocyanins can enter into cells and activate the Nrf2/HO-1 pathway, conferring resistance against oxidative damage and increasing antioxidant defence [152], and can interact with other natural antioxidants, such as other phenolic compounds, carotenoids, and vitamins, which also increase their ability to relieve oxidative stress [134]. Nevertheless, and although no study has revealed toxicity on humans, it is imperative to conduct further in vivo studies in order to reveal the safe dosage of phenolics intake, including of anthocyanins and anthocyanidins, as they can act as pro-oxidants in some conditions (e.g., a basic pH, high concentrations of transition metal ions, and the presence of oxygen, among others) [153].

### 6.3. Anti-Inflammatory Properties

Anthocyanins also possess anti-inflammatory capacity. Inflammatory conditions happen in response to pathogens that were not removed (e.g., autoimmunity) or due to an inadequate long-term response to stimuli (e.g., allergies). They are characterized by oedema, redness, pain, fever, function losses, and larger amounts of pro-inflammatory cytokines (e.g., tumour necrosis factor (TNF)-*α*, interleukin (IL)-6, and 1*β*) and nitric oxide radicals [154]. Therefore, it is important to be treated as soon as possible given their involvement in chronic disorders, such as asthma, obesity, gout, diabetes, cancer, atherosclerosis, and neurological pathologies.

Among the anthocyanins, cyanidin and delphinidin 3-*O*-glucosides already exhibited potential to reduce the C-reactive protein levels by 77% in human liver cancer HepG2 cells, and vascular cell adhesion molecule-1 secretion in endothelial cells by 47%, at concentrations of 50 µg/mL compared to the non-exposed cells group [13]. Additionally, delphinidin, petunidin, and malvidin 3,5-diglucosides also revealed capacity to inhibit nitric oxide release, and IL-6, IL-1*β*, and TNF-*α* in lipopolysaccharide (LPS)-induced RAW264.7 macrophages at concentrations of 80 µg/mL [152]. Anthocyanin extracts from raspberries (concentrations of 100, 150, and 200 µg/mL) also showed an ability to reduce the expression levels of cyclooxygenase-2 (COX-2), inducible nitric oxide synthase (iNOS), and IL 1*β* and IL-6, and to suppress AP-1 signalling and nuclear factor kappa B (NF-kB) pathways. Additionally, it was also verified that they can decrease IKK, IkBa, p65, and JNK phosphorylation, avoid p65 nuclear translocation in LPS/IFN-γ-stimulated RAW 264.7 macrophage cells [155], and inhibit lipoxygenase activity, at concentrations of 10, 25, and 50 µg/mL [154]. Similar results were obtained concerning the anthocyanins isolated from strawberries and blackberries at a concentration of 50 µg/mL [156], and purple sweet potato at 200 µg/mL [152]. Besides, anthocyanin-fractions extracted from berries also showed potential to reduce TNF-*α*, IL-8, and Regulated upon Activation, Normal T Cell Expressed and Presumably Secreted (RANTES) at doses between 10 and 25 µg/mL in human bronchial epithelial BEAS-2B normal cells treated with LPS [157]. These results are in line with those recently described by Chen et al., who proved that an anthocyanin-rich extract from mulberry fruits, at a concentration of 50 mg/mL, can reduce the IL-6, iNOS, phospho-p65, and phospho-IκB*α* pro-inflammatory markers, and increase the IL-10 concentration in RAW264.7 macrophage cells stimulated with LPS over 24 h of exposure [128]. 

Enriched fractions of anthocyanins from raspberries already showed anti-inflammatory effects in dextran sulphate sodium-induced acute colitis in mice. Animals ingested 20 mg/kg of the extract for 10 days. At the end of the study, blood samples were collected, and mice were sacrificed for histological assessment. The results were compared in the control group; i.e., rats only received a normal diet. The obtained data revealed that a diet rich in anthocyanins contributed to improve colitis damage, by enhancing pro-inflammatory markers reduction and controlling weight [155]. Similar data were observed by Pereira et al. [158], who’s study was based on the administration of blueberries also in 2,4,6-trinitrobenzenesulfonic acid-induced colitis rat models, at concentrations of 10 mg/kg over 8 days of treatment, compared to an untreated group. This study proved the ability of anthocyanins to downregulate iNOS, inhibit COX-2 expression, decrease leukocyte infiltration, and increase antioxidant defence in the colon. Tart cherry anthocyanins (400 mg/kg) administrated for 3 days also showed an ability to suppress inflammation-induced pain in rats, essentially due to their ability to inhibit cyclooxygenase-mediated synthesis of prostaglandins [159]. Furthermore, Kim et al. revealed that 5 mg of encapsulated anthocyanins per day can decrease COX-2, NOS3, IL-1*β,* and TNF-*α* inflammatory cytokines in mice brain [121]. Another study related to anthocyanins extracted from rice (at 25 µg/mL) proved that they can increase type I collagen gene expression and protect skin fibroblasts against H_2_O_2_ damage, essentially by inhibiting IκBα phosphorylation, NF-κB activation, and IL-6 production, over 24 h of exposure [160].

Regarding human studies, 150 hypercholesterolemic subjects who ingested 320 mg anthocyanin capsules, rich in cyanidin 3-*O*-glucoside and delphinidin 3-*O*-glucoside, daily for 24 weeks showed lower levels of C-reactive protein, vascular cell adhesion molecule-1, and plasma IL-1*β* levels (−20, −13 and −4%, respectively) when compared to the untreated group. No changes were detected regarding TNF-*α* concentrations, which indicate that the intervention was safe [13]. Jacob et al. reported that the consumption of 280 g of cherries (28 mg per 100 g of fresh weight) twice a day can also reduce the plasma C-reactive protein and nitric oxide levels by 29.4% and 16.8%, respectively, when compared with the baseline. A decrease in plasma urate (−14.5%) and increments in ascorbic acid (+9%) were also verified, highlighting the anti-gout effects of cherries. This trial was composed of ten healthy women who consumed cherries after an overnight fast [161]. These data are in accordance with Kelley et al., who conducted a study involving 18 healthy men and women that were subjected to the daily consumption of 280 g cherries for 28 days. The results revealed a decrease in C-reactive protein, nitric oxide, and RANTES at percentages of −25, −18 and −21%, respectively. No changes were observed in IL-6, neither in the high-, low-, and very-low-density lipoprotein cholesterols nor triglycerides, which can be considered evidence regarding the security of this supplementation dose [162]. Furthermore, ten healthy adult volunteers who drank 10 g of *Hibiscus sabdariffa* diluted in water showed lower monocyte chemoattractant protein-1 (−23.2%) levels, an important biomarker considering the evaluation of inflammatory disorders, after 3 h of their consumption compared to the placebo group [163]. Additionally, a pilot study, composed of 13 patients with mild to moderate ulcerative colitis, consumed 160 g of blueberries (4 trays per day), corresponding to 95 g dw (around 600 g of fresh fruit) during six weeks, revealed that 63.4% of patients achieved remission of the inflammatory bowel disease [164]. In another study, sixteen volunteers were subjected to the consumption of a 250 mL dose of anthocyanin-rich plum juice; the results obtained revealed a decrease in the C-protein reactive, IL-6, IL-1*β*, and TNF-*α* concentrations of−22, −7, −9 and −4%, respectively. These data were observed 4 h after the intake relative to the placebo group [165]. 

Besides the mentioned, anthocyanins also facilitate muscle recovery after intense exercise, attenuating its damage and inflammation [149,150,151]. Indeed, sixteen active students who drank 480 mL of blackcurrant nectar for eight consecutive days revealed lower levels of creatine kinase, a marker of muscle damage, and IL-6, 48 h post physical activity [149]. These results were similar as those obtained by Hurst et al. also based on the consumption of blackcurrant nectar (~240 mg total anthocyanins). In this work, a 5-week randomized placebo-controlled pilot trial composed of 36 volunteers revealed that the group who consumed this juice showed lower levels of pro-inflammatory TNF-*α* and IL-6 molecules and increments in anti-inflammatory IL-10 cytokines [151]. Furthermore, twenty runners who consumed 30 mL of tart cherry juice during 5 days before and 48 h after a marathon run also showed reduced levels of inflammatory markers (IL-6, uric acid, and C-protein reactive) compared to the placebo group [150].

These effects are closely linked to the ability of anthocyanins to prevent CD40 activation, a member of the tumour necrosis factor receptor superfamily [166]. Additionally, they also can inhibit the cyclooxygenase isoenzymes, COX-1 and COX-2, and interfere with the MAPK cascade [121]. Indeed, and through an in vitro cyclooxygenase inhibitory assay with enzymes from ram seminal vesicles, anthocyanins extracted from sweet cherries and raspberries, at a concentration-dose of 125 µg/mL, showed more potential to inhibit COX-1 activity (47.4 and 54.3%, respectively) than ibuprofen (39.8%) and naproxen (41.3%) at 10 µM [167].

### 6.4. Anticancer Properties

Cancer causes more deaths than strokes and coronary disorders and it is characterized by uncontrolled cell growth and proliferation [8]. Anthocyanins have demonstrated capacity to inhibit the initiation, promotion, and progression of types of cancer, such as human colon [134,168], liver and bladder [169,170], breast [171], brain [172], renal and skin [168], gastric [141], and thyroid [173] cancers, mainly due to their antioxidant properties and capacity to interfere with PI3K/Akt [174].

The capacity of anthocyanins to inhibit the growth of metastatic cells is not surprising. Indeed, the enriched fraction of anthocyanins from sweet cherries already showed an ability to inhibit human adenocarcinoma Caco-2 cells growth (inhibitory IC_50_ value of 667.84 µg/mL), causing necrosis at concentrations superior to 400 µg/mL, after 24 h of treatment [134]. Similar effects were verified using coloured phenolics from pollen, but at a higher concentration (10 mg/mL) [175]. Concentrations higher than 500 µg/mL of anthocyanin-rich extracts from the *Myrtaceae* family also showed an ability to reduce the proliferation of human colon adenocarcinoma HT-29 cells, mainly by arresting the G2/M phase, hence causing cell apoptosis in comparison with the control group over 24 h of treatment [176]. It was also already verified that anthocyanin-rich extracts from pomegranate at 50 µg/mL can reduce by 10% the growth of human bladder cancer T24 cells growth after 48 h of exposure, in comparison with untreated cells [170]. Additionally, and after 2 days of an incubation assay, anthocyanins from cherries also showed capacity to interfere with MDA-MB-453 and MDA-MB-231 breast cancer cell lines growth, revealing IC_50_ values of 45 and 149 µg/mL, respectively, without toxicity to MCF-10A normal breast cells [171]. Blackberry and raspberry fruits at 250 µg/mL also revealed effectiveness in stopping cancer cell growth, inhibiting human colon HCT-116, breast MCF-7, lung NCI-H460, and gastric AGS tumour cell lines by 50, 24, 54, and 37%, respectively, after 24 h of exposure [141]. Furthermore, 10 µg/µL of anthocyanins from mulberries revealed capacity to suppress thyroid SW1736 and HTh-7 cancer cell proliferation, by inducing apoptosis and autophagy-dependent cell death, and inhibiting protein kinase B and ribosomal protein S6 activation, after 72 h of treatment [173]. On the other hand, açaí extracts rich in anthocyanins showed the ability to suppress C-6 rat brain glioma cell growth, showing cell viability reductions of 62, 45, and 38% at concentrations of 50, 100, and 200 µg/mL, respectively, after 48 h of treatment [172]. More recently, Vilkickyte et al. reported that anthocyanins isolated from lingonberry fruits can suppress the viability of malignant melanoma IGR39 and renal CaKi-1 cancer cells, displaying IC_50_ values of 200 and 400 µg/mL, respectively [168]. 

Focusing on in vivo studies, the daily administration of berries (0.5 mg/kg body weight) in rats with induced oesophageal carcinoma showed an ability to reduce cancer growth by 22% over 20 weeks of treatment, when compared to the control group [177]. Adding 10% dietary freeze-dried berries to the standardized diet of rats also showed capacity to inhibit induced oesophagus cancer by 30–60% and colon cancer by up to 80% when compared to the control, over 25 weeks of treatment [178]. 

Furthermore, a recent study revealed that anthocyanins from blueberry extracts showed the capacity to prevent the formation and growth of colorectal cancer in azoxymethane/dextran sodium sulphate-treated Balb/c mice, which ingested a diet containing 10% of the extract during 9 weeks of treatment, compared with the control [179]. Anthocyanins from *Vitis coignetiae* Pulliat also showed the potential to inhibit the tumorigenicity in mice infected with Hep3B human hepatocellular carcinoma cells (5 µg/g of animal per day) [180].

On the other hand, berry anthocyanins displayed anticarcinogenic effects on lung cancer progression in rat xenograft models (reductions around 40%) treated with a diet supplemented with 7.5%, after 6 weeks of the intervention [181]. The supplementation during 1 month with 0.5% of cyanidin and peonidin 3-*O*-glucosides also exhibited the capacity to reduce Lewis lung carcinoma cells in mice [182].

In addition, the administration of delphinidin (2 mg, three times a week) in athymic nude mice implanted with PC3 cells resulted in significant inhibition of prostate tumour growth, essentially by reducing the expression of NF-κB/p65, Bcl2, Ki67, and PCNA after 8 weeks of treatment [183]. An anthocyanin mixture extracted from black soybean also inhibited xenograft growth of prostate cancer in mice treated with a daily oral anthocyanin (8 mg/kg) after 14 weeks of intervention [184]. 

Besides, the daily administration of anthocyanin-rich extracts from black rice (100 mg/kg, during 28 weeks) in mice xenografted with human tumour models showed the ability to reduce breast cancer growth, causing reductions around 41% [185], mainly due to their ability to inhibit growth factor receptor 2, a gene overexpressed in this type of cancer [186]. Blackthorn fruits at 0.2 mg daily (5 days a week, for one month) already showed potential to slow down tumour growth xenografts in immunodeficient mice injected with human colon HCT116 cancer cells [187]. Anthocyanins from black rice already showed potential to increase immune responses in murine leukaemia cells, at concentrations higher than 50 mg/kg over 3 weeks of treatment, promoting CD3 (T cell), CD19 (B cell), CD11b (monocyte), and macrophage phagocytosis, and decreasing the NK cell activity when compared to the untreated control group [188].

Regarding skin cancer, the administration of anthocyanins extracted from *Sorbus aucuparia* (5 mL/kg for 11 days) in rats showed potential to reduce melanoma [189]. Furthermore, Lee et al. reported that anthocyanins extracted from aronia conjugated with fucoidan, a natural polysaccharide extracted from seaweed, can prevent the development of induced skin tumour in mice when administered twice a week at a concentration of 900 mg/kg dose during 22 weeks [174].

Although there are no relevant studies regarding the anti-cancer effects on humans, it was already verified that the consumption of 20 g of black raspberry powder for between 2 and 4 weeks (three times per day) by Barrett’s oesophagus patients, a precursor lesion for oesophageal tumour, can reduce the proliferation rates and CD 105-stained blood vessels, and increase apoptosis in colon tumours [190]. 

The ability of anthocyanins to inhibit the initiation and development of a tumour is closely associated with their ability to increase antioxidant defences, exert anti-inflammatory actions, and interfere with the ERK, JNK, PI3K/Akt, MAPK, and NF-κB pathways, as well as being associated with their regulated proteins and influence on the estrogenic/antiestrogenic levels [180,183]. Furthermore, these flavonoids compounds also showed apoptotic effects since they can activate the caspase cascade, reduce the mitochondria membrane potential, and modulate the cytochrome C and aromatase activities [185,191]. 

### 6.5. Neurological Properties

Knowing neurological pathologies are closely related to oxidative stress and inflammatory levels, it is not surprising that the consumption of secondary metabolites from plants, such as anthocyanins, can reduce their occurrence.

Anthocyanin-enriched fractions extracted from berries at 20 µg/mL already showed capacity to reduce nitric oxide, reactive oxygen, and carbonyl species, as well as the H_2_O_2_ levels and apoptotic protease caspase-3/7 activity in BV-2 microglia cells in about 20% relative to untreated cells. They also showed an ability to diminish anti-glycation and anti-Aβ A aggregation [192]. Furthermore, anthocyanin-rich extracts from blueberries at 0.01 µg/mL, together with cyanidin 3-*O*-glucoside, cyanidin 3-*O*-sophoroside, delphinidin 3-*O*-glucoside, and malvidin 3-*O*-glucoside at 1 µM, also revealed an ability to protect dopaminergic neurons from the primary cells of Parkinson’s disease against rotenone toxicity, after 72 h of treatment, essentially by attenuating mitochondrial dysfunction [193].

Besides, it was already reported that anthocyanins (ingestion of 100 mg/kg for 10 months) can restore the ion pump activities in rats experimentally demyelinated with ethidium bromide, by diminishing the proinflammatory mediators’ secretion and oxidative-stress levels and restoring the IL-10 levels [131]. Additionally, anthocyanins isolated from black bean at concentrations varying from 100 and 200 µg/mL (over 12 h of treatment) proved their ability to protect the hippocampal neurons of mice against toxicity induced by kainic acid, mainly thanks to their capacity to reduce the reactive oxygen species levels, caspase-3, AMPK activation, and mitochondrial cytochrome-c release into the cytoplasm, and in attenuating the Ca^2+^ perturbations, losses of mitochondrial integrity, and Bax accumulation [194]. Kim et al. revealed that 5 mg (administered during 14 days) of anthocyanins encapsulated in gold nanoparticles can cross the blood–brain barrier and reduce the amyloid-β_1-42_ plaques and neuro-apoptotic markers (anti-Bax, anti-Bcl2, anti-caspase-3, anti-cytochrome c, and anti-P-JNK) by inhibiting the *ρ*-JNK/NF-*κ*B/ρ-GSK3*β* pathway in mice brain [121].

Concerning human assays, a 12-week randomised study involving 49 older adults with mild-to-moderate dementia who drank 200 mL anthocyanin-rich cherry juice showed improvements in verbal fluency, learning, memory, and cognition when compared to the untreated group [14]. Similar neurological improvements were observed after 12 weeks of blueberry concentrate supplementation in healthy older adults, who drank 30 mL of blueberry juice compared to the placebo [15]. Whyte et al. also conducted a study involving one hundred and twenty-two older adults, which consumed 16 capsules per week over 3 months of blueberry-rich extracts. As expectable, better cognitive tasks regarding executive function, working memory, and episodic memory were registered in comparison to the placebo [195].

As mentioned before, the neuroprotective effects showed by anthocyanins are mainly due to their ability to cross the blood–brain barrier and protect neurons and glia cells from oxidative damage induced by reactive species and free radicals, reduce the inflammatory cytokines and *β*-amyloid concentrations, and to suppress NF-*k*B, the Nf E2-related factor-2 (Nrf-2) signalling pathway, COX, and caspase activities [119]. Furthermore, anthocyanins also can raise gene expression, GTPase activity, and detoxifying enzyme amounts, and therefore meliorate cerebrovascular and peripheral blood flow [196].

### 6.6. Anti-Diabetic and Anti-Obesity Effects

Diabetes mellitus, with type 2 standing out, affects several people worldwide, being associated with obesity, oxidative stress, inflammatory events, and cardiovascular risk [197]. Given the resistance developed to the current pharmaceutical drugs administrated, the ingestion of anthocyanin-rich products has been encouraged. In fact, anthocyanins already showed mechanisms capable to diminish insulin resistance, hyperglycaemia, proinflammatory cytokines, and radical species; inhibit gluconeogenesis and the action of the *α*-amylase and *α*-glucosidase carbohydrate-hydrolysing enzymes; and, consequently, restore the glucose levels, incentive insulin secretion, and pancreatic *β*-cells proliferation [198,199].

Among the anthocyanins, cyanidin 3-*O*-glucoside already showed potential to inhibit pancreatic α-amylase activity, revealing an IC_50_ value of 24.4 µM [200]. Anthocyanin-enriched fractions from sweet cherries also showed the capacity to inhibit the *α*-glucoside action, showing to be three times more efficient than the acarbose control, which is the current pharmaceutical drug used to treat type 2 diabetes, but whose intake cause several gastrointestinal side effects [134]. Besides, anthocyanins from *Cornus kousa* displayed anti-angiogenic effects in the pre-adipocyte cell line 3T3-L1s, suppressing lipogenesis and adipogenesis in comparison with untreated cells after 8 days of exposure [201].

The oral administration of acarbose combined with cyanidin 3-*O*-rutinoside (30 mg/kg) also revealed an ability to alleviate postprandial hyperglycaemia and inhibit intestinal *α*-glucosidase in normal rats over three hours of intervention, as compared with the control group [202]. Besides, it was already documented that the glucoside of cyanidin can also meliorate diabetes-related endothelial dysfunction in mice that received a 6 mg/kg diet for 8 weeks, by stimulating adiponectin expression and improving flow-mediated dilation [203]. On the other hand, black currant anthocyanins showed the ability to reduce body weight gain and glucose levels and improve insulin sensitivity in mice that were fed with 8 mg/day of anthocyanins for 8 weeks. Converted into human equivalent doses, these ones are estimated to be 1.5 g/day of total anthocyanins for an average adult [56]. Furthermore, the daily administration of anthocyanins from *Hibiscus sabdariffa* L. (300 mg/kg) in obese-hypercholesterolemic rats revealed the capacity to reduce their weight, as well as to improve their lipid profile and liver enzymes action over 3 weeks of treatment in comparison to the group that did not receive the diet [198]. Blueberry anthocyanins also showed anti-obesity effects. Indeed, their administration at doses of 200 mg/kg during one month showed the ability to reduce the body weight of obese C57BL/6 mice by 19.4% and also diminish the glucose and pro-inflammatory levels, improve the lipid profile, and suppress the peroxisome proliferator-activated receptor-γ, FAS genes, and fatty acid synthesis over a 16-week treatment in contrast to the untreated group [204]. These results were similar as those reported by Kwon et al. and Wu et al. regarding the oral ingestion of anthocyanins from black soybean, black rice, and purple corn by obese rats [16,205].

Regarding human studies, it was already reported that twenty dyslipidaemic children and adolescents who daily ingested 50 g of cornelian cherry fruits, during 6 weeks, showed better lipid, apolipoprotein and vascular inflammation profiles and lower levels of LDL and triglycerides, intracellular adhesion molecule-1 and vascular cell adhesion molecule-1 (−10, −13, −30 and −25%, respectively). Higher levels regarding apolipoprotein A (+11.6%) and a diminished content of apolipoprotein B (−13.6%) were also verified [197].

The anthocyanins potential to be used as novel therapeutic agents against diabetes and obesity is fundamentally attributed to their chemical structure, which gives them inhibitory effects on the carbohydrate-hydrolysing enzymes’ action and capacity to interact in a competitive and/or non-competitive mode with the enzymes’ substrate and create hydrophobic bonds with these enzymes, thereby discontinuing their act and retard carbohydrate absorption [134,200]. Furthermore, anthocyanins also revealed the capacity to raise GLUT 4 membrane translocation expression and upregulate the signalling pathway of the peroxisome proliferator-activated receptors, encouraging adipocyte glucose uptake and improving the lipid profile [10].

### 6.7. Cardiovascular Properties

Cardiovascular pathologies are the principal cause of morbidity and mortality worldwide. They are intimately associated with the adoption of unhealthy behaviours, such as smoking, excessive alcohol intake, and other risk factors, including metabolic syndrome [33]. Several studies indicate that the daily intake of anthocyanin-rich fruits, vegetables and beverages can attenuate, or even prevent, the occurrence of coronary events, since they can modulate lipid metabolism, fat deposition, and endothelial function, as well as reduce blood pressure and vascular adhesion molecules expression. These activities are intimately associated with their antioxidant and anti-inflammatory characteristics [206].

Particularly, cyanidin 3-*O*-glucoside and delphinidin 3-*O*-glucoside at concentrations of 50 µM showed the ability to reduce in vitro platelet aggregation and lysosome secretion, essentially by reducing *β*-thromboglobulin, serotonin ATP, platelet factor 4, CD63, and transforming growth factor *β*1 secretion after 40 min of exposure [207]. Moreover, it was already documented that cyanidin 3-*O*-glucoside can stimulate adiponectin expression and improve flow-mediated dilation in human adipose tissues and human aortic endothelial cells treated with 50 µM of cyanidin 3-*O*-glucoside after 24 h of exposure [203]. Blueberry anthocyanidins (cyanidin, delphinidin, and malvidin) are also reported to inhibit human umbilical vein endothelial cell-induced tube formation in a co-culture with fibroblasts at concentrations ranging from 0.3 and 10 µM [208].

Moreover, 50 µg/mL of anthocyanin-enriched fractions from strawberries already showed capacity to reduce the triglycerides and low-density lipoprotein (LDL) contents around 17% and 23%, respectively, in HepG2 cells after 24 h of treatment [169]. Additionally, 50 mg/mL of encapsulated anthocyanins extracted from blueberries and black currants already showed the potential to inhibit platelet aggregation by suppressing P-selectin expression and stimulate the thromboxane A2 pathway [209]. 

Concerning in vivo trials, it was already reported that blueberry anthocyanin-enriched extracts can attenuate cyclophosphamide-induced cardiac damage in rats, treated with 80 mg/kg during four weeks by ameliorating the arterial blood pressure, heart rate, and activities of the heart enzymes, and improving cardiac dysfunction, left ventricular hypertrophy, and fibrosis. Additionally, they also showed the potential to prevent cardiomyocyte apoptosis [206]. 

Furthermore, 31 pre-hypertensive men who ingested a single dose of 640 mg anthocyanins daily for 4 weeks revealed higher levels of high-density lipoprotein (HDL) concentrations and lower concentrations of triglycerides [210]. Moreover, the consumption of two capsules (80 mg each per day during 28 days) rich in anthocyanins already showed the ability to reduce monocyte-platelet aggregate formation (−39%), procaspase activating compound-1 (−10%), P-selectin (−14%), and inhibit platelet endothelial cell adhesion molecule-1 expression (−14%) [17]. In another work, the authors reported that the consumption of 250 g per day of blueberry powder, for 6 weeks, by 13 volunteers showed an ability to increase natural killer cells and reduce arterial stiffness in sedentary males and females, mainly by diminishing diastolic pressure [211]. This fact is in accordance with Whyte et al. [195]. These authors conducted a study involving 122 volunteers who consumed 16 capsules per week of blueberry-rich extracts (100 mg of anthocyanins/capsule) over six months. Habanova et al. reported a study of 36 volunteers who consumed 150 g of frozen blueberries 3 times a week, for 6 weeks. The obtained results revealed lower levels of LDL glucose albumin, aminotransferase, alkaline phosphatase, and γ-glutamyltransferase, accompanied by higher levels of HDL when compared to the baseline [212]. These data are in line with that reported by Arevström et al., who conducted a study based on the daily ingestion of 40 g of blueberries powder (equivalent to 480 g fresh blueberries) over 8 weeks of treatment [213]. Additionally, reductions around 5% in systolic and diastolic blood pressures were also observed in older adults with mild-to-moderate dementia who drank 200 mL anthocyanin-rich cherry juice for 12 weeks [156]. Similar results were reported by Draijer et al., who conducted a human trial involving 60 subjects who consumed 6 grape-wine extract capsules (~247.3 mg anthocyanins per capsule) every day over ten weeks. Additionally, a reduction in endothelin-1 by 10% was also found compared to the control group [214]. The obtained results are in line in those obtained by Igwe et al., who conducted a pilot cross-over study involving 24 adults, but who focused on the intake of 100 mL of anthocyanin-rich plum juice three times a day [215]. Besides, the dietary supplementation of 500 mg of aronia extracts over 12 weeks also revealed the capacity to modulate the lipid profile by diminishing the fasting plasma total cholesterol (−8%), LDL, (−11%) and LDL receptor protein in peripheral blood mononuclear cells (−31.9%), as compared to placebo group. Significant correlations between the obtained results and cyanidin 3-*O*-galactoside and peonidin 3-*O*-galactoside contents were found [216]. Similar results were reported by Bakkar et al., who conducted a study involving twelve overweight middle-aged men that consumed 226 mg of encapsulated anthocyanins from tart cherries daily for 28 days [217]. Furthermore, blueberries also showed the potential to reduce the peak postprandial glucose levels and extend the postprandial glucose response in 17 healthy young adults who consumed a range of doses varying from 310 and 724 mg of freeze-dried wild blueberry powder, 2 h following their consumption [218]. Beyond that, the oral ingestion of 320 mg of anthocyanins isolated from berries, over 12 weeks, by hypercholesterolemic individuals also showed the potential to increase brachial artery flow-mediated dilation (+10%) and HDL amounts (+12.8%), and diminish serum soluble vascular adhesion molecule-1, triglycerides, and LDL concentrations (−11.6, −4.1 and −10.0%, respectively) [219], and also glycated haemoglobin (HbA1c) (−0.14%) [118]. Additionally, it has also been mentioned that the intake of four anthocyanins capsules per day (total of 320 mg/day) can reduce plasma *β*-thromboglobulin, soluble P-selectin, platelet factor 4, and transforming growth factor *β*1 levels in hypercholesterolaemic patients for 24 weeks of treatment as compared with the baseline [207]. These positive effects are correlated with the delphinidin 3-*O*-glucoside and cyanidin 3-*O*-glucoside concentrations [219]. Similar effects were reported in adults overweight and obese who drank 200 mL blood orange juice twice a day for 2 weeks [19]. The consumption of one 350 mg capsule every 8 h for 2 months also showed the potential to meliorate the lipid profile of hyperlipidaemic patients, by reducing the total cholesterol, triglyceride, and LDL by −27.6, −19.2%, and −26.3%, respectively, and raising HDL by +37.5% when compared with the baseline [220]. On the other hand, seven days’ intake of 600 mg per day of blackcurrant extracts containing 210 mg of anthocyanins showed the potential to increase vasodilation, total haemoglobin, and cardiac output, and decrease the muscle oxygen saturation, which is beneficial in improving exercise performance [221].

Therefore, the regular consumption of anthocyanin-rich foods and beverages can contribute to reduce the risk of cardiomyopathies, coronary problems, and ischemia [8,33]. These effects are due to the antioxidant and anti-inflammatory effects of anthocyanins, given that nitric oxide radicals and pro-inflammatory cytokines are critical factors in cardiovascular diseases. In fact, these compounds can inhibit p38 mitogen-activated protein kinases, c-Jun N-terminal kinase activation, and the PI3K/Akt signalling pathways, consequently attenuating eNOS phosphorylation and cGMP production, interrupt MAPK activation, and the recruitment of TNF-receptor-associated factors-2 in lipids, in the way of protecting endothelial cells from CD-40 proinflammatory effects [166].

### 6.8. Eye Improvement

In vivo and in vitro evidence also revealed that anthocyanins can improve the eyes and thus vision, owing to their ability to increase blood circulation in retina capillaries and the production of retinal pigments, which, in turn, improve night vision and protect eyes from oxidative damage, diabetic retinopathy, and molecular degeneration [8].

Anthocyanins isolated from blackberries at 100 µg/mL showed potential to protect human adult retinal pigment epithelial ARPE-19 cells against oxidative stress induced by H_2_O_2_, by increasing the activity of heme-oxygenase-1 and glutathione S-transferase-pi antioxidant enzymes after 24 h of exposure [222]. Similar data were reported by Sundalius [223] regarding blueberry anthocyanins, over 7 days of treatment.

Concerning in vivo studies, delphinidin 3-*O*-rutinoside at 10 µM already showed an ability to relax the bovine ciliary smooth muscle through activation of the endothelin-1 receptor and NO/cGMP pathway, inhibiting myosin light chain phosphorylation, hence causing relaxation [224]. Additionally, anthocyanins extracted from black currants showed the potential to inhibit vitreous-chamber depth enlargement, and the axial and ocular lengths of chicks when compared to the controls, over 3 days of treatment at a dose concentration of 200 mg/kg [225].

Focusing on human assays, a double-blind, placebo-controlled, crossover study involving healthy volunteers revealed that the daily intake of six capsules rich in blackcurrant anthocyanins at a concentration of 50 mg can improve dark adaptation, video display terminal work-induced transient refractive alteration, and asthenopia symptoms (visual fatigue) in comparison to the placebo [226]. In another study based on the ingestion of anthocyanins from blueberry, the results revealed that the patients with normal-tension glaucoma presented vision improvement, not only due to anthocyanins’ oxidative properties but also owing to their ability to increase the blood circulation [18].

Given that, it was already reported that intact anthocyanins can pass through the blood-aqueous barrier and blood-retinal barrier in rats and rabbits, being widely distributed in ocular tissues [224]. This evidence, together with their antioxidative effects and blood circulation improvements, suggests that anthocyanins can be considered a potential drug therapy for treating ophthalmological diseases, such as myopia and glaucoma [8,18].

## 7. Conclusions

Anthocyanins are the coloured compounds largely found in nature, for which evidence indicates that their regular consumption offers several health benefits to human health, mainly due to their ability to reduce free radicals, reactive species, and pro-inflammatory markers. These abilities can counteract oxidative stress levels, avoid the development of inflammatory processes, and protect human organs and cell components against damage, and thus confer protection at distinct levels. Thus, anthocyanins’ structure, biochemistry, and encapsulation have been deeply studied in order to increase their use, stability, and consequent bioavailability and action. Until now, and although more in vivo and clinical trials are needed, evidence suggests that anthocyanins are promising candidates for the engineering of new pharmaceutical drugs, and can be used as an alternative or as an adjuvant therapy capable to attenuate or prevent the occurrence of many disorders, including diabetes, cancer, and cardiovascular and neurological pathologies. In fact, their use in pharmaceutical products, nutraceuticals, foods, and as food colourants is increasing worldwide.

## Figures and Tables

**Table 1:** Concentration of anthocyanins in fresh weight (FW) and dried weight (DW) in fruits, beverages, and vegetables.

Source	Maximum Anthocyanin Amount in FW (mg C3G/100 g)	Maximum Anthocyanin Amount in DW (mg C3G/100 g)	Dominant Anthocyanins	Reference
Fruits				
Açaí	282.5–303.7		Cy 3,5-hexose pentose, Cy 3-*O*-glucoside, Cy 3-*O*-rutinoside, Pg 3-*O*-glucoside, Pn 3-*O*-glucoside, Pn 3-*O*-rutinoside, Cy 3-(acetyl)hexose	[51]
Acerola	6.5–8.4		Cy 3-rhamnoside, Pg 3-rhamnoside Cy, Pg	[51]
Apple	30.07–71.49 *		Cy 3-galactoside, Cy 3-*O*-glucoside, Cy 3-arabinoside, Pn 3-galactoside, Cy 7-arabinoside, Cy3-xyloside	[46,52]
Blackberries	70.3–201	909.3	Cy 3-*O*-glucoside, Cy 3-*O*-rutinoside, Cy 3-xyloside, Cy 3-malonylglucoside, Cy 3-dioxalylglucoside	[53,54]
Black currants	218.93	32,300 ^1^	Dp 3-*O*-glucoside, Dp 3-*O*- rutinoside, Cy 3-*O*-glucoside, Cy 3-*O*- rutinoside	[55,56]
Blueberries	406.90	1160^1^	Dp 3-*O*-galactoside, Dp 3-*O*-glucoside, Cy-3-*O*-galactoside, Dp 3-*O*-arabinoside, Cy-3-glucoside, Pt-3-galactoside, Cy 3-*O*-arabinoside, Pt 3-*O*-glucoside, Pn 3-*O*-arabinoside, Mv 3-*O*-galactoside, Mv 3-*O*-glucoside	[55,57]
Chokeberries	357.20		Cy 3-*O*-galactoside, Cy 3-arabinoside, Cy 3-*O*-glucoside, Cy xyloside	[55,58]
Cranberries	40.7–207.3 ^1^		Cy 3-*O*-galactoside Cy 3-*O*-glucoside, Cy arabinoside, Pn galactoside, Pn 3-*O*-glucoside, Pn arabinoside	[59]
Elderberries	317.51	408.6–1066.6	Cy 3-*O*-glucoside, Cy 3-*O*-sambubioside	[55,60]
Fig	0.3–10.9	4.6–83	Cy 3-*O*-glucoside, Cy 3-*O*-rutinoside, Cy 3-sambubioside-5-glucoside, Cy 3,5-diglucoside	[61,62]
Grapes	38.70–186.02 ^1^	135,960–533,630 ^1^	Cy, Dp, Mv, Pn, Pt 3-*O*-glycosides; Mv, Pn, Pt 3-*O*-coumarylglucosides	[63,64]
Haskaps	449–697	2273	Cy 3,5-di-glucoside; Cy 3-galactoside, Cy 3-*O*-glucoside, Cy 3-*O*-rutinoside, Pg 3-*O*-glucoside; Pn 3-*O*-glucoside	[65,66]
Nectarine	0.22		Cy 3-*O*-glucoside, Cy 3-*O*-rutinoside	[46,67]
Plums	7.4–36.6 ^1^		Cy 3-xyloside, Cy 3-*O*-glucoside, Cy 3-*O*-rutinoside, Pn 3-*O*-rutinoside, Pn 3-*O*-glucoside, Cy 3-galactoside, Cy 3-(6’’-acetoyl)glucoside	[46,68]
Pomegranate	1500–2000		Dp 3,5-diglucoside, Cy 3-*O*-glucoside, Cy 3,5-diglucoside, Pg 3-*O*-glucoside, Pg 3,5-diglucoside	[69,70]
Peaches	0.27–2.50	0.28–15.34 ^1^	Cy 3-*O*-rutinoside and glucoside	[71,72]
Red cabbages	109–185	1111–1780	Cy 3-diglucoside-5-glucoside, Cy 3-(sinapoyl)(sinapoyl)-diglucoside-5-glucoside, Cy 3-(*ρ*-coumaroyl)-diglucoside-5-glucoside	[73,74]
Red currants	19.78	149.91 ^1^	Cyanidin-3-*O*-sambusoside, Cy 3-*O*-glucoside, Cy 3-*O*-rutinoside	[55,75]
Red pears	1.2–12.0 ^1^		Cy 3-*O*-galactoside, Cy 3-*O*-glucoside, Cy pentoside, Cy 3-*O*-arabinoside, Cy 3-*O*-rutinoside	[76]
Red raspberries	23.17–68.0	260.9–571.8	Cy 3-*O*-sophoroside, Cy 3-*O*-(2’’-*O*-glucosyl)rutinoside, Cy 3-*O*-glucoside, Cy 3-*O*-rutinoside, Cy 3-*O*-(2’’-O-xylosyl)rutinoside, Pg 3-*O*-sophoroside, Pg 3-*O*-glucoside, Cy 3,5-*O*-diglucoside	[53,55,77]
Strawberries	20–60 ^1^	31.9–315.2 ^2^	Pg 3-*O*-glucoside, Pg 3-*O*-glucoside, Cy 3-*O*-glucoside, Cy 3-*O*-rutinoside, Pg 3-*O*-glucoside, Pg 3-*O*-rutinoside, Pg 3-(malonoyl)glucoside, Pg 3-(6’’-acetoyl)glucoside	[46,78,79]
Sweet cherries	2.06–462.77 ^1^	107.70–218.36 ^1^	Cy, Dp, Pg, Pn 3-*O*-rutinosides and glucosides, Cy 3-coumaroyl-diglucoside, Cy 3-*O*-sambubioside, Cy 3-5-diglucoside, Cy 3-sophoroside Cy 3-*O*-arabinoside Mv 3-*O*-glucoside-acetaldehyde	[40,80,81]
Tamarillo		29.70–486.84 ^1^	Dp 3-*O*-rutinoside, Cy 3-*O*-rutinoside, Cy 3-*O*-glucoside, Pg 3-*O*-rutinoside	[82]
Tart cherries	65.06–82.40	114.59	Cy, Cy 3-*O*-sophoroside, Cy 3-glucosylrutinoside, Cy 3-*O*-glucoside, Cy 3-*O*-rutinoside, Pn 3-*O*-rutinoside	[55,83]
Tomato	7.1 ^1^	5.48–29.86 ^3^	Dp glycoside, Dp rutinoside, Dp *ρ*-coumaroyl-rutinoside Mv glycoside, Mv rutinoside, Mv *ρ*-coumaroyl-rutinoside-glycoside, Pt rutinoside, Pt *ρ*-coumaroyl-rutinoside, Pt *ρ*-coumaroyl-rutinoside-glycoside	[84,85]
Vegetables				
Black carrot	22.45 *	1.74–4.54 ^1^	Cy 3-(*ρ*-coumaroyl)-diglucoside-5-glucoside	[47,86]
Eggplant	6.31	138 ^4^	Dp 3-(*ρ*-coumaroylrutinoside)-5-glucoside, Dp 3-*O*-glucoside, Dp 3-glucosyl-rhamnoside, Pt -3-*O*-rutinoside, Cy -3-*O*-rutinoside	[49,50]
Purple sweet potato	42.37 *		Pn 3-*O*-sophoroside-5-*O*-glucoside, Pn 3-*O*-glucoside, Cy 3-*ρ*-hydroxybenzoylsophoroside-5-glucoside, Pn 3-*ρ*-hydroxybenzoylsophoroside-5-glucoside, Cy 3-caffeoylsophoroside-5-glucoside, Pn 3-caffeoylsophoroside-5-glucoside, Cyanidin-3-caffeoyl- *ρ*-hydroxybenzoylsophoroside-5-glucoside, Pn 3-dicaffeoylsophoroside-5-glucoside, Pn 3-caffeoyl-*ρ*-hydroxybenzoylsophoroside-5-glucoside, Pn 3-caffeoy-feruloylsophoroside-5-glucosie	[47]
Red Chicory	39.20 *		Cy 3-*O*-glucoside, Cy 3-*O*-(6”-malonyl-glucoside)	[47]
Red onion	29.99		Cy 3-*O*-glucoside, Cy 3-*O*-laminaribioside, Cy 3-(6’’-malonyl-glucoside), Cy 3-(6”-malonyl- laminaribioside), Cy 3-xylosylglucosylgalactoside, Dp 3,5-diglycosides	[47,87]
Beverages				
Blackberry juice	12.3–107		Cy 3-*O*-glycoside, Cy 3-*O*-rutinoside, Cy 3-xyloside, Cy malonylglucoside, Cy dioxalylglucoside	[54]
Pomegranate juice	7.2–20 ^1^		Cy 3-*O*-glucoside, Cy 3,5-diglucoside, Dp 3,5-diglucoside, Cy 3,5-diglucoside, Pg 3,5-diglucoside, Dp 3-glucoside, Cy 3- *O*-glucoside; Pg 3-*O*-glucoside	[88]
Red wine	32.71–87.17 ^1^		Cy, Dp, Mv, Pn, Pt 3-*O*-glycosides, Pn 3-*O*-acetylglucoside, Mv 3-*O*-acetylglucoside, Mv 3-*O*-coumarylglucoside, Pn 3-*O*-*ρ*-coumarylglucoside;	[48,89]
Tart cherry juice		72.2	Cy 3-sophoroside, Cy 3-glucosylrutinoside, Cy 3-*O*-glucoside, Cy 3-*O*-rutinoside, Cy, Pg, Pn 3-*O*-glucoside	[90]

FW, fresh weight; DW, dry weight; Cy, cyanidin; Dp, delphinidin; Mv, malvidin; Pg, pelargonidin; Pn, peonidin; Pt, petunidin. * mg cyanidin 3-galactoside equivalent per 100 g. ^1^ Total amount (weight) of anthocyanins identified by HPLC method. ^2^ mg pelargonidin 3-glucoside equivalent per 100 g. ^3^ mg malvidin 3-rutinoside equivalent per 100 g. ^4^ Delphinidin 3-glucoside equivalent per 100 g.

**Table 2:** Human studies considering the pharmacokinetic parameters of anthocyanins, after ingestion of common foods and beverages rich in this type of compound.

Intake	*n* ^(a)^	Total Anthocyanins Intake	C_max_ ^(b)^	t_max (h)_ ^(c)^	AUC ^(d)^	Urinary Excretion (%)	Reference
Foods							
Blueberries (100 g)	5	1200 mg	13.1 ng/mL	4			[57]
Elderberries (12 g)	4	720 mg	97.4 nmol/L	1.2 h			[94]
Red raspberries (300 g)	9	292 µmol	0.1–180 nmol/L	1–1.5		0.007% (1–1.5 h)	[77]
Beverages							
Red wine (300 mL)	6	218 mg		6		1.5–5.1% (12 h)	[89]
Red grape juice (400 mL)	9	Cy 3-*O*-glucoside	0.42 ng/mL	0.5	0.60 ng × h/mL (3 h)	0.09% (7 h)	[97]
		Dp 3-*O*-glucoside	6.12 ng/mL	0.5	11.9 ng × h/mL (3 h)	0.20% (7 h)	
		Mv 3-*O*-glucoside	48.8 ng/mL	0.5	71.7 ng × h/mL (3 h)	0.18% (7 h)	
		Pn 3-*O*-glucoside	27.3 ng/mL	0.5	49.7 ng × h/mL (3 h)	0.29% (7 h)	
		Pt 3-*O*-glucoside	16.1 ng/mL	0.5	31.5 ng × h/mL (3 h)	0.32% (7 h)	
		Σ = 283.5 mg	100.1 ng/mL	0.5	168.4 ng × h/mL		
Black currant juice (150 mL)	8	Cy 3-*O*-glucoside: 0.165 mg	5.0 nmol/L	1.34	11.0–19.6 ng × h/mL (4 h)	0.060% (8 h)	[96]
		Cy 3-*O*-rutinoside: 1.24 mg	46.3 nmol/L	3.45	19.6–24.9 ng × h/mL (4 h)	0.098% (8 h)	
		Dp 3-*O*-glucoside: 0.488 mg	22.7 nmol/L	4.19	11.0–16.3 ng × h/mL (4 h)	0.066 (8 h)	
		Dp 3-*O*-rutinoside: 1.68 mg	73.4 nmol/L	3.18	16.3–24.9 ng × h/mL (4 h)	0.11% (8 h)	
Açaí Juice (7 mL/kg of body weight)	12	165.9 mg/L	1138.51 ng/L	2	3314.04 ng × h/L (12 h)		[11]
Cranberry Juice (480 mL)	15	Cy 3-*O*-galactoside (18.7 mg)	1.38 nmol/L	1.27	3.91 nmol × h/L (4 h)	0.007% (4 h)	[98]
		Cy 3-*O*-glucoside (1.58 mg)	0.93 nmol/L	1.13	1.99 nmol × h/L (4 h)	0.007% (4 h)	
		Cy 3-*O*-arabinoside (16.47 mg)	3.61 nmol/L	1.47	9.16 nmol × h/L (4 h)	0.010% (4 h)	
		Mv 3-*O*-glucoside	0.56 nmol/L	0.93	1.25 nmol × h/L (4 h)		
		Pn 3-*O*-galactoside (30.83 mg)	4.64 nmol/L	1.47	12.00 nmol × h/L (4 h)	0.015% (4 h)	
		Pn 3-*O*-glucoside (5.85 mg)	0.71 nmol/L	1.40	1.85 nmol × h/L (4 h)	0.029% (4 h)	
		Pn 3-*O*-arabinose (21.03 mg)	1.78 nmol/L	1.27	4.13 nmol × h/L (4 h)	0.010% (4 h)	
		Σ = 94.47 mg					
Tart cherry juice (60 mL)	12	62.47 mg/L	2.75 µg × h/mL	1	106.4 µg × h/mL		[99]
Grape/blueberry juice (330 mL)	10	3,4-dihydroxybenzoic acid	7.6 nmol/L	1	568 nmol × min/L		[100]
		Cy 3-*O*-glucoside	0.10 nmol/L	1	6 nmol × min/L		
		Dp 3-*O*-glucoside	0.18 nmol/L	1.1	10 nmol × min/L		
		Mv 3-*O*-glucoside	1.5 nmol/L	1.1	103 nmol × min/L		
		Mv 3-*O*-glucuronide	1.1 nmol/L	2	114 nmol × min/L		
		Pn 3-*O*-glucuronide	1.1 nmol/L	1.8	114 nmol × min/L		
		Pn 3-*O*-glucoside	1.7 nmol/L	1	52 nmol × min/L		
		Pt 3-*O*-glucoside	0.8 nmol/L	1	12 nmol × min/L		
		Σ = 841 mg/L	1.21 nmol/L				
Orange juice	18	53.09 mg/L	0.63 nmol/L	0.96	8.99 nmol × h/L (8 h)	43–53% (2 h)	[101]
Chokeberry juice (0.8 mg/kg of body weight)	13		32.7 nmol/L	1.3	109.4 nmol × h/L (1 h)	0.25 (24 h)	[102]
Strawberry juice (34.7 mg)	14	Cy 3-*O*-glucoside: 7.8 µmol	0.6 nmol/L	2.1	1.7 nmol × h/L (10 h)		[95]
		Pg glucuronide	38.0 nmol/L	1.7	123.8 nmol × h/L (10 h)		
		Pg-3-*O*-glucoside: 58.8 µmol	5.2 nmol/L	1.3	15.0 nmol × h/L (10 h)		
		Pg 3-*O*-rutinoside: 9.7 µmol	0.4 nmol/L	1.9	1.4 nmol × h/L (10 h)		
		Σ = 76.6 µmol					

^(a)^ Number of participants. ^(b)^ Maximum concentration in plasma. ^(c)^ Time to reach C_max_ in plasma. ^(d)^ Area under the curve, which describes the exposure of a compound over a set period of time.

## Data Availability

Data sharing not applicable.
